# The Use of Blended Teaching in Higher Medical Education during the Pandemic Era

**DOI:** 10.1155/2022/3882975

**Published:** 2022-11-14

**Authors:** Xue-Tao Fu, Yi Hu, Bing-Chun Yan, Yun-Gen Jiao, Shi-Jun Zheng, Ying-Ge Wang, Jiang-Yun Zhang, Zheng-Bing Wang

**Affiliations:** ^1^Department of Neurology, The Affiliated Hospital of Yangzhou University, Yangzhou 225000, Jiangsu, China; ^2^Department of Education, The Affiliated Hospital of Yangzhou University, Yangzhou 225000, Jiangsu, China

## Abstract

**Objective:**

This study aims to compare the effect of blended teaching and traditional teaching in higher medical education during the pandemic era.

**Methods:**

Taking the teaching of neurology as an example, 293 Yangzhou University Clinical Medicine 2016 undergraduate students were selected as the research subjects, and were randomly divided into 2 groups a blended teaching group (*n* = 148) and a traditional teaching group (*n* = 145), and received blended teaching and traditional teaching, respectively. The blended teaching was based on a Massive Open Online Course, problem-based learning, and case-based learning and supplemented by Tencent video conferences, QQ messaging groups, and other auxiliary teaching tools. At the end of the course, the teaching effect and satisfaction rate were evaluated through theory assessment, practical skills assessment, and an anonymous questionnaire survey.

**Results:**

There were significant differences in theoretical achievements (81.83 ± 6.23 vs 76.79 ± 6.87, *P* < 0.001) and practical skill achievements (84.74 ± 6.50 vs 78.48 ± 6.53, *P* < 0.001). In addition, significant differences in all aspects of satisfaction rate were observed between the two groups (all *P* < 0.001).

**Conclusion:**

Blended teaching is beneficial to students' learning and stimulates their enthusiasm, cultivates clinical thinking ability, and improves teaching quality. Thus, it has played a positive role in the reform of higher medical teaching during the pandemic era.

## 1. Introduction

A novel coronavirus outbreak occurred in Wuhan at the end of 2019 and quickly swept across China. Novel coronavirus pneumonia not only poses a major threat to human health but also has a huge impact on all areas of life. Facing the great challenges of Novel coronavirus pneumonia, universities and colleges have adopted online teaching to achieve the goal of “stop lessons but not teaching, stop lessons but not learning [1],” and, as a result, a Massive Open Online Course (MOOC) has become the focus of attention with their unique advantages. MOOC is an open online network course produced with modern information technology [2]. The original design concept was to take teaching resources, educators, teaching designers, and teaching implementers as a whole, build an interactive learning platform through network information technology, and realize the wide dissemination of online teaching content. It plays an important role in the teaching reform of colleges and universities [3, 4]. With the gradual stabilization of the pandemic situation in China, students have returned to school to resume classes. But it does not mean that the risk is completely eliminated. For a long time to come, the whole country may be in a state of normalized pandemic prevention and control. If care is not taken, the pandemic will return. Therefore, during the pandemic era, how to give consideration to the prevention and control of the pandemic situation and, at the same time, the optimization of teaching has become an urgent problem facing educational institutions.

Blended teaching, as a new educational model in recent years, has achieved good results in different fields [5, 6]. In order to meet the needs of higher medical education reform during the pandemic era, and taking the teaching of neurology as a pilot scheme, we established a blended teaching mode. It is based on MOOC, problem-based learning (PBL) [7], and case-based learning (CBL) [8] and supplemented by Tencent conferences, QQ groups, and other auxiliary teaching tools.

## 2. Data and Methods

### 2.1. Subjects

Using the cluster sampling method, 293 undergraduates majoring in clinical medicine from six classes of Yangzhou University in 2016 (University of fourth grade) were selected as the research subjects. This study was conducted from September to December 2020. Inclusion criteria were as follows: (1) undergraduates majoring in clinical medicine in 2016; (2) all ethnic groups are Han nationality, proficient in using Mandarin; (3) Proficiency in using computers and smartphones; (4) no visual, auditory, and language barriers. Exclusion criteria were as follows: (1) repeated students; (2) transferred students; (3) foreign students; (4) minority students. These subjects were randomly divided into two groups a blended teaching group (three classes, *n* = 148) and a traditional teaching group (three classes, *n* = 145).

### 2.2. Teaching Methods

The teachers, syllabus, and class hours of the two groups were the same.

For the blended teaching group, MOOC was used as a basis, and PBL and CBL teaching methods were also employed, with Tencent video conferences and QQ messaging groups being used as auxiliary teaching tools. The details of the teaching program were as follows: (1) MOOC teaching: the teachers would release an announcement before class to inform the students of the content to be learned in advance so that the students could have a targeted preview. In order to improve student participation, teachers inserted classroom tests into the teaching contents of each chapter. The test types included single-choice questions, multiple-choice questions, blank-filling questions, and judgment questions. Through the backstage management page, teachers could find out their students' video viewing situation, learning duration, and test results at any time. This meant they could monitor student participation and learning and allowed them to give targeted feedback and supervision. (2) PBL teaching [9, 10]: according to the requirements of the syllabus, the teachers compiled the teaching plan and devised activities of all types, moving from surface to deep questions, from linked to progressive sessions; students retrieved literature, accessed information, collated the content, made records, and actively participated in the discussion, based on their own analysis and thinking of the problems and issues they were presented with. (3) CBL teaching [11]: based on the teaching syllabus, the teachers collected relatively rare but typical cases found in diagnosis and treatment work, compiled and produced complete teaching cases, and organized students to discuss and study the problems related to the points of knowledge in each case. (4) QQ groups: before giving a course, teachers set up a class QQ messaging group for corresponding courses and assigned group administrators (such as a monitor and study committee) to assist in the management and organization of class students' course learning. Via a group announcement, the teacher released the course information, teaching schedule, syllabus and examination arrangement, uploaded videos, documents, courseware, homework, and other teaching materials, and carried out learning surveys, online discussion, feedback, and communication through the QQ groups. (5) Tencent conferences [12]: any material and content missing from the MOOCs were supplemented by Tencent video conferences, where key points and difficult content in the course were analyzed and explained in depth, and online Q and A counseling was possible.

In the traditional teaching group, teachers adopted face-to-face classroom teaching. In line with the neurology syllabus, the teachers compiled a teaching plan, worked out the teaching schedule and class arrangements and taught theoretical knowledge with the use of slides, videos, blackboard writing, and other traditional tools.

### 2.3. Observation Indexes

At the end of the course, the theoretical and practical skills of the two groups of students were assessed, and the results of the teaching were comprehensively evaluated through examination results. The theoretical assessment was conducted by 2 teachers in the teaching and research section independently based on the content required by the syllabus, which would be collected and randomly selected by the Academic Affairs Office to form the final exam paper. The repetition rate of the questions used in the final exam and the usual test was not more than 10%. Practical skills assessment included neurological examination and lumbar puncture. The total score of the theoretical assessment and practical skill assessment was 100. At the same time, a self-developed anonymous questionnaire (Appendix 1) was used to evaluate student satisfaction and recognition of blended teaching and traditional teaching. The questionnaire covered five areas: course content, time schedule, curriculum design, classroom atmosphere, and teaching mode. The students declared themselves as being very satisfied, relatively satisfied, dissatisfied, or very dissatisfied, which scored one, two, three, and four points, respectively, with respect to each area.

### 2.4. Student and Public Involvement

Using the cluster sampling method, 293 undergraduates majoring in clinical medicine from six classes of Yangzhou University in 2016 (University of fourth grade) enrolled in this study. The use of the data including data protection issues was discussed with the students. Information on the register was made available to the public on a website.

### 2.5. Statistical Methods

Data were statistically analyzed using the statistical software SPSS 23.0. Measurement data were expressed as mean ± standard deviation (*x* ± SD), and compared between groups using a *t*-test. The inspection level was set at *α* = 0.05, and *P* < 0.05 was considered statistically significant.

## 3. Results

### 3.1. Baseline Characteristics

There were 78 males and 70 females in the blended teaching group; the subjects ranged from 21 to 23 years of age, the mean age being 22.13 ± 0.51 years old. There were 76 males and 69 females in the traditional teaching group; these subjects ranged from 21 to 23 years of age as well, with the mean age being 22.21 ± 0.49 years old. The differences in age, gender, and other clinical data between the two were not statistically significant (*P* > 0.05), and, hence, the two groups were comparable.

### 3.2. A Comparison of Examination Results between the Two Groups of Students

**Figure 1:**
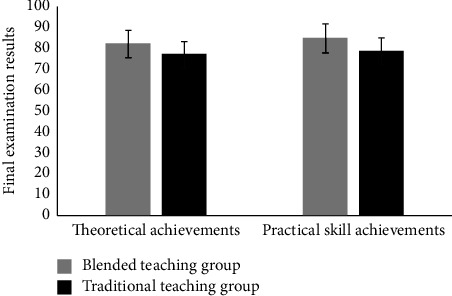
Comparison of final examination results between the blended teaching group and traditional teaching group.

A comparative analysis of the theoretical examination and practical skills examination between the blended teaching group and the traditional teaching group showed that the theoretical examination of the blended teaching group was 81.83 ± 6.23, which was significantly higher than the traditional teaching group of 76.79 ± 6.87, and the difference was statistically significant (*P* < 0.01). The practical skills examination of the blended teaching group was 84.74 ± 6.50, which was significantly higher than 78.48 ± 6.53 in the traditional teaching group, and the difference was statistically significant (*P* < 0.01) (Table 1, Figure 1).

### 3.3. A Comparison of the Satisfaction Rate between the Two Groups of Students

**Figure 2:**
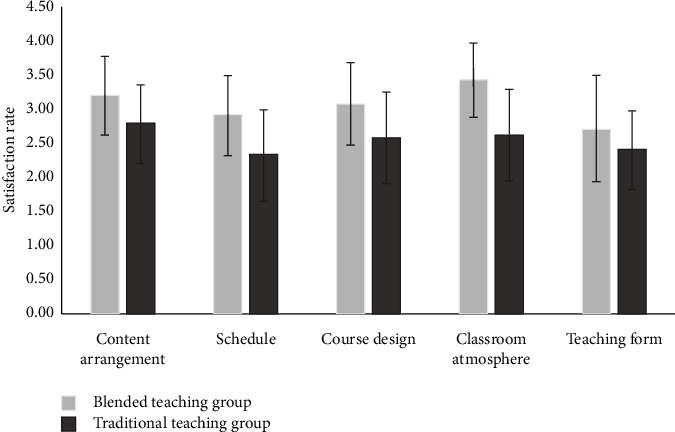
Comparison of satisfaction rate between the blended teaching group and traditional teaching group.

Comparing differences between the two groups of students in content arrangement, schedule, course design, classroom atmosphere, and teaching form, the results showed that the satisfaction of the blended teaching group in the above aspects (3.18 ± 0.58, 2.89 ± 0.58, 3.07 ± 0.60, 3.42 ± 0.54, and 2.70 ± 0.77) were significantly higher than the traditional teaching group (2.77 ± 0.58, 2.32 ± 0.67, 2.57 ± 0.67, 2.60 ± 0.67, and 2.40 ± 0.57), and the difference was statistically significant (*P* < 0.001) (Table 2, Figure 2).

## 4. Discussion

Higher medical education during the pandemic era is faced with many difficulties and challenges. During their isolation at home, students experienced varying degrees of irritability, anxiety, and panic. Furthermore, remaining at home for a long time disrupted their regular learning patterns, and lacking supervision from teachers, over time, meant some poorly self-disciplined students became lazy and demotivated, resulting in a decline in learning quality. During the pandemic era, the following challenges exist: how to connect online teaching and offline teaching smoothly; how to coordinate and complement the contents of online teaching and offline teaching; when students fail to keep up with the pace of online teaching for various reasons, how to make up for it in the later teaching process.

In order to address the above problems and challenges, a diverse blended teaching mode was established at Yangzhou University, to effectively make up for the limitations of a single teaching method and to meet the requirements of the training of high-quality medical personnel during the pandemic era, and this study looked at how effective the blended teaching approach was in achieving its objectives compared to the more traditional approach to teaching.

MOOCs were first proposed by Canadian scholars George Siemens and Stephen Daones in 2008 [13]. As an open online course, a MOOC constructs a public learning platform through information technology, integrates and shares various teaching resources, so as to realize the wide dissemination of teaching content, and plays a decisive role in the teaching of colleges and universities in the Internet era [14–17]. Taking advantage of the flexibility and immediacy of MOOCs, students can go beyond the limits of space and time, flexibly arrange learning time according to their own situations, and use the fragmented time to learn online at any time and place, avoiding learning interruption caused by network congestion and signal instability. For the key and most difficult content, Students also have the opportunity to learn at their own pace and deepen their understanding by reviewing the content as often as they wish some students who have the time and energy can also learn other knowledge related to the subject through the shared resources of the MOOC platform, so as to expand their academic vision and broaden the scope of their knowledge.

However, a MOOC has certain limitations, which is especially obvious in clinical medicine education. For example, there is not much interaction between teachers and students, it is difficult to realize individualized teaching, and it is difficult to play the main role of students. There is also a lack of a sound evaluation mechanism as well as supervision and management of the teaching process, and students cannot achieve a good combination of theoretical knowledge and clinical practice [18].

In order to make up for the above limitations, in line with the characteristics of the neurology course, we combined PBL and CBL teaching methods together with a MOOC, so as to stimulate student initiative, enthusiasm, and creativity in learning, improve student concentration on the course content, and cultivate students' clinical thinking ability, in order to optimize the teaching results.

PBL is a problem-based, teacher-oriented, and student-centered group discussion teaching method [19]. It can improve students' ability to analyze, make judgments, use independent thinking [20], and also helps students to apply theoretical knowledge to clinical practical problems, so as to effectively consolidate their knowledge [21, 22]. CBL is a “case-based, problem-based, student-centered, teacher-led” teaching method [23]. Neurology has a wide spectrum of diseases, various clinical manifestations, and complicated and changeable conditions, so it is very difficult for students to acquire the perceptual knowledge of clinical rare diseases only by theoretical explanation. CBL teaching, with its unique teaching method, effectively makes up for this deficiency and can make abstract and boring theoretical knowledge vivid, and guide students through finding out and thinking about problems and solving them, in the process of case analysis.

The combination of PBL and CBL gives students a chance to participate in the discussion and develop their self-confidence. In addition, rather than just paying attention to a teacher the students can impart knowledge to others, and their clinical thinking ability and on-site responsibility can improve as well as their language expression [24, 25].

Through the practical exploration of blended teaching, comprehensive reform and innovation in the teaching methods, course content, and teaching modes of the neurology course took place at Yangzhou University. The approach encapsulates the concept of seeing students as the main resource, and teachers as guides and facilitators. Students generally showed a keen interest and gave a high degree of recognition to this new teaching mode. The investigators realize that each teaching method has its own advantages and disadvantages, and no single teaching method can meet the growing needs of medical education reform. In today's mobile Internet era, with the rapid development of network platforms and electronic information technology, medical education is changing from the traditional classroom teaching mode to a diversified blended teaching mode. During the pandemic era, the use of network platforms and media technology to skillfully combine a variety of teaching methods can effectively mobilize student initiative, stimulate enthusiasm for learning, and lead to the cultivation of high-quality comprehensive medical talent.

Our research still has limitations. This study only selected students in one grade in 2016 (because the neurology course is set in the fourth grade of the University). It was a single-center study and had not been compared with other courses or students in other grades. For the shortcomings of this research, we would have the opportunity to conduct multicenter research jointly with other grades, disciplines, and other institutions in the future to further verify the results.

At present, while the situation of pandemic prevention and control is still grim, we should keep pace with the times, strive to be more flexible, and use diversified teaching methods in order to adapt to the developments and changes in the teaching situation during the pandemic era, so as to make a modest contribution to the national antipandemic work. In the future, the combination of various teaching methods can deal with the impact of the epidemic more flexibly. Each teaching method has its own advantages and disadvantages, and a single teaching method can no longer meet the development needs of medical education reform. The integration of various teaching methods can effectively stimulate students' learning enthusiasm and initiative and help to cultivate high-quality comprehensive medical talents.

## Figures and Tables

**Table 1:** Comparison of final examination results between the blended teaching group and traditional teaching group (*x* ± SD, points).

	Blended teaching group (*n* = 148)	Traditional teaching group (*n* = 145)	*T*	*P*
Theoretical achievements	81.83 ± 6.23	76.79 ± 6.87	6.579	<0.001
Practical skill achievements	84.74 ± 6.50	78.48 ± 6.53	8.235	<0.001

**Table 2:** Comparison of satisfaction rate between the blended teaching group and traditional teaching group (*x* ± SD, points).

	Blended teaching group (*n* = 148)	Traditional teaching group (*n* = 145)	*t*	*P*
Content arrangement	3.18 ± 0.58	2.77 ± 0.58	6.065	<0.001
Schedule	2.89 ± 0.58	2.32 ± 0.67	7.737	<0.001
Course design	3.07 ± 0.60	2.57 ± 0.67	6.746	<0.001
Classroom atmosphere	3.42 ± 0.54	2.60 ± 0.67	11.541	<0.001
Teaching form	2.70 ± 0.77	2.40 ± 0.57	3.833	<0.001

## Data Availability

The datasets used and/or analyzed during the current study are available from the corresponding author upon reasonable request.

## Supplementary Materials

Supplementary Materials Teaching satisfaction Questionnaire of Yangzhou University Medical College.
