# Supplementary material for: The Use of Blended Teaching in Higher Medical Education during the Pandemic Era
Source: Int J Clin Pract. 2022 Nov 14;2022:3882975. doi: 10.1155/2022/3882975 (PMC9678442; doi:10.1155/2022/3882975)
Supplement: Supplementary Materials — Teaching satisfaction Questionnaire of Yangzhou University Medical College. [file 3882975.f1.docx]

| Appendix 1: Teaching satisfaction Questionnaire of Yangzhou University Medical College | | | | | |
| --- | --- | --- | --- | --- | --- |
|  | Very satisfied | Relatively satisfied | Dissatisfied | Very dissatisfied | Score |
| Content Arrangement | 4 | 3 | 2 | 1 |  |
| Schedule | 4 | 3 | 2 | 1 |  |
| Course Design | 4 | 3 | 2 | 1 |  |
| Classroom atmosphere | 4 | 3 | 2 | 1 |  |
| Teaching form | 4 | 3 | 2 | 1 |  |
